# Thermochemical Study of CH_3_NH_3_Pb(Cl_1__−*x*_Br*_x_*)_3_ Solid Solutions

**DOI:** 10.3390/ma15217675

**Published:** 2022-11-01

**Authors:** Maxim Mazurin, Angelika Shelestova, Dmitry Tsvetkov, Vladimir Sereda, Ivan Ivanov, Dmitry Malyshkin, Andrey Zuev

**Affiliations:** Institute of Natural Sciences and Mathematics, Ural Federal University, 19 Mira St., Ekaterinburg 620002, Russia

**Keywords:** mixed halide perovskites, thermochemistry, mixing enthalpy, molecular dynamics

## Abstract

Hybrid organic–inorganic perovskite halides, and, in particular, their mixed halide solid solutions, belong to a broad class of materials which appear promising for a wide range of potential applications in various optoelectronic devices. However, these materials are notorious for their stability issues, including their sensitivity to atmospheric oxygen and moisture as well as phase separation under illumination. The thermodynamic properties, such as enthalpy, entropy, and Gibbs free energy of mixing, of perovskite halide solid solutions are strongly required to shed some light on their stability. Herein, we report the results of an experimental thermochemical study of the CH_3_NH_3_Pb(Cl_1−*x*_Br*_x_*)_3_ mixed halides by solution calorimetry. Combining these results with molecular dynamics simulation revealed the complex and irregular shape of the compositional dependence of the mixing enthalpy to be the result of a complex interplay between the local lattice strain, hydrogen bonds, and energetics of these solid solutions.

## 1. Introduction

Organic–inorganic halide perovskites APbX_3_ (A = methylammonium, formamidinium cations, X = Cl, Br, I anions) belong to the promising class of compounds with a wide range of potential applications [1]. Among them, particular attention has been drawn to the field of solar energy conversion [2]. However, one of the main problems hindering the development and commercialization of the devices based on halide perovskites is their well-known relative instability under ambient conditions against moisture, oxygen, heating, etc. [3]. Since halide perovskites are believed to be entropy stabilized compounds [4], due to their relatively low formation enthalpy and large formation entropy from binary halides, the obvious solution proposed to improve the stability is to increase the entropy of perovskite halides even more through the formation of solid solutions [5,6], i.e., compounds with a partial substitution of ions in the A, Pb, or X sublattices or even simultaneously in all of them, in an attempt to achieve a compromise between the stability and desired physical properties [2,7,8,9,10].

However, the stability of all the compounds including halide perovskite solid solutions is governed, neglecting the kinetic limitations, by thermodynamics which has not been studied experimentally for most of the perovskite halide solid solutions, except for the CsPb(I_1−*x*_Br*_x_*)_3_ (x = 0, 1/3, 1) [11] and A_1__−x_(en)_x_Pb_1−0.7x_X_3−0.4x_ (A = methylammonium, formamidinium; en = ethylenediammonium; X = Br, I) [12]. For the vast majority of the known perovskite halide solid solutions, the conclusions on their stability are based mainly on experimental trial-and-error tests, which may be subject to kinetic hindrance, or on the results of density functional theory (DFT) calculations. In general, for compounds containing elements of sufficiently different chemical natures (oxides, halides, nitrides, etc.), such calculations do not reach a sufficient level of accuracy (so-called chemical accuracy ≈1 kcal·mol^−1^ or 4.184 kJ·mol^−1^) in predicting formation enthalpies even when employing computationally expensive approaches [13,14,15]. As an example of the trial-and-error studies, the work [16], which reports the behavior of CH_3_NH_3_Pb(Cl_1−*x*_Br*_x_*)_3_ or CH_3_NH_3_Pb(I_1−*x*_Br*_x_*)_3_ solid solutions, can be mentioned. It was shown in [16] that under illumination, both solid solutions undergo a phase separation which was found to be (at least partially) reversible. Various theoretical approaches [17,18,19,20] were used to explain this puzzling behavior, but the results obtained look to be ambiguous. For instance, the phase diagram of the CH_3_NH_3_Pb(I_1−*x*_Br*_x_*)_3_ system suggested in [17] showed the wide range of solid solutions that are unstable under ambient conditions and should decompose spontaneously to the Br- and I-rich phases. These results were criticized [21] since the reversible phase separation observed in [17] is inconsistent with the suggested spontaneous spinodal decomposition.

Furthermore, the enthalpy contribution to the Gibbs free energy of mixing evaluated by the ab initio approaches is typically treated as a simple uniform curve (symmetric or asymmetric), having no features and corresponding to the regular/subregular solution behavior [17,18,19]. The same is also true for the mixing entropy which is usually treated as a configuration one, neglecting the potential additional contributions such as, for example, vibrational, defect, etc.

However, the compositional dependence of the excess thermodynamic properties may be more complex, as shown by the results of the molecular dynamics simulation for the title CH_3_NH_3_Pb(Cl_1−*x*_Br*_x_*)_3_ solid solutions [19]. This contradicts the results of the quantum chemistry calculations mentioned above.

Therefore, the thermodynamic stability of perovskite halide solid solutions is still an open question, and experimental thermochemical studies are strongly required to address it. Herein, we report the results of such studies combined with molecular dynamics simulation performed for CH_3_NH_3_Pb(Cl_1−*x*_Br*_x_*)_3_ solid solutions to reveal the complex interplay between their energetics, local lattice strain, and hydrogen bonding.

## 2. Materials and Methods

All reagents used in this work were taken “as-received”, i.e., without a further purification. A full list of reagents with their chemical grades is given in Table S1.

The methylammonium halides CH_3_NH_3_X (X = Cl, Br) were synthesized according to the following reaction (the subscript ‘aq’ denotes the aqueous solution state):

CH_3_NH_2(aq)_ + HX_(aq)_ = CH_3_NH_3_X_(aq)_. (1)

An aqueous solution of methylamine (38.5 mass %) and a corresponding solution of hydrohalic acid diluted 1:1 by distilled water were used as the starting reagents. Both solutions were slowly mixed with each other under continuous stirring and cooling. The acid for the reaction was taken in slight excess with respect to the stoichiometric quantity. The resulting solutions of methylamine hydrohalides were evaporated at *T* ≈ 353 K until the beginning of crystallization and then cooled to room temperature. Precipitated crystals were isolated by suction filtration and carefully washed with ethanol.

Lead (II) bromide was prepared according to the following reaction (the subscript ‘s’ denotes the solid state of material):

Pb(CH_3_COO)_2(aq)_ + 2 HBr_(aq)_ = PbBr_2(s)_↓ + 2 CH_3_COOH_(aq)_. (2)

A total of 10.3 g of Pb(CH_3_COO)_2_·3H_2_O were dissolved in 10 mL of distilled water under continuous stirring and heating at *T* ≈ 333 K. In addition, the as-prepared solution was filtered through a paper filter and heated to boiling. Then, 10 mL of concentrated hydrobromic acid (~20% excess over stoichiometric amount) were added dropwise to the boiling solution. The white precipitate of PbBr_2_ immediately formed. The solution with the precipitate was slowly cooled to room temperature and stored for about 1–2 h for the recrystallization of the precipitate, which was further isolated by suction filtration and thoroughly washed with ethanol.

All the prepared binary halides were dried in an oven at *T* ≈ 353 K, accurately ground, and then stored in a vacuum desiccator under phosphorus oxide P_2_O_5_.

The mixed halide perovskites were prepared by solid state synthesis according to the following reactions (the subscript ‘s’ denotes the solid state of material):

(1 − 3*x*) CH_3_NH_3_Cl_(s)_ + PbCl_2(s)_ + 3*x* CH_3_NH_3_Br_(s)_ = CH_3_NH_3_Pb(Cl_1−*x*_Br*_x_*)_3(s)_, (3)

(2 − 3*x*) CH_3_NH_3_Cl_(s)_ + 0.5 PbCl_2(s)_ + 0.5 PbBr_2(s)_ + (3*x* − 1) CH_3_NH_3_Br_(s)_ = CH_3_NH_3_Pb(Cl_1−*x*_Br*_x_*)_3(s)_, (4)

(3 − 3*x*) CH_3_NH_3_Cl_(s)_ + PbBr_2(s)_ + (3*x* − 2) CH_3_NH_3_Br_(s)_ = CH_3_NH_3_Pb(Cl_1−*x*_Br*_x_*)_3(s)_, (5)

where Equation (3) was used for compositions with *x* ≤ 0.3, Equation (4) for 0.4 ≤ *x* ≤ 0.6, and Equation (5) for *x* ≥ 0.7.

**Figure 1:**
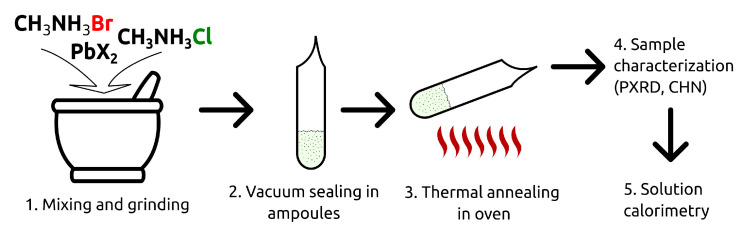
The schematic representation of the solid solutions synthesis process.

Schematically, the synthetic procedure is presented in Figure 1. The stoichiometric amounts of the binary halides were mixed, carefully ground in an agate mortar, and placed into evacuated and hermetically sealed glass ampoules. The ampoules were heated in an oven at *T* = 423−463 K for a period of 2–5 days (no significant differences in the result of the synthesis were noticed within the indicated temperature range). After an exposure at the specified temperature, the ampoules were quickly ejected and cooled to room temperature. The as-obtained samples were stored in a vacuum desiccator under P_2_O_5_.

The phase purity of all the prepared powders (binary halides and perovskite solid solutions) was analyzed by the powder X-ray diffraction (PXRD). The PXRD data were collected at *T* = (298 ± 3) K (expanded uncertainty, level of confidence 95%) using an Inel Equinox 3000 (Inel SAS, Artenay, France) diffractometer with Cu Kα radiation in the 2Θ range from 10° to 90°.

Indexing and Rietveld refinement of the PXRD patterns were carried out using a GSAS-II software package [22]. The crystal structures of CH_3_NH_3_PbCl_3_ [23] and CH_3_NH_3_PbBr_3_ [24] were used as the starting models with occupancies of Cl and Br atoms corresponding to the chemical composition of a solid solution.

The content of C, H, and N was determined using a PE-2400 CHN analyzer (Perkin Elmer, Waltham, MA, USA).

The solution enthalpies of the mixed halide perovskites in dimethylsulfoxide (DMSO) at *T* = (298.15 ± 0.04) K (expanded uncertainty, level of confidence 95%) were measured using a home-built isoperibolic calorimeter, described in detail elsewhere [25]. The reliability of the calorimeter was confirmed by measuring the dissolution enthalpy of potassium chloride KCl as a reference substance. The measured solution enthalpy, corrected to an infinite dilution, was found to be (17.211 ± 0.069) kJ·mol^−1^ (expanded uncertainty, 95% confidence level), which is in a good agreement with the recommended reference value (17.241 ± 0.018) kJ·mol^−1^ (expanded uncertainty, 95% confidence level) [26].

DMSO was used as a suitable calorimetric solvent due to the high solubility of lead halide perovskites and their solid solutions in it. The molality of the solutions after the calorimetric experiments was ca. 2 × 10^−3^ mol·kg^−1^. The samples were dissolved at least 3 times, and the resulting enthalpy was averaged. The ambient pressure during the measurements was (100 ± 4) kPa (expanded uncertainty, level of confidence 95%).

**Figure 2:**
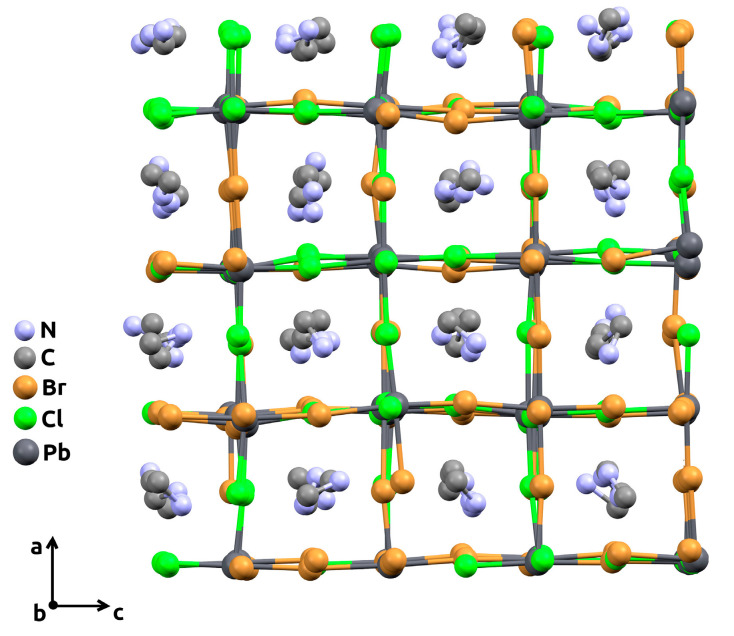
The 4 × 4 × 4 supercell (view along the crystallographic *b-*axis) with mi*x*ed chlorine-bromine content (*x* = 0.5), obtained after molecular dynamics simulation procedure. Hydrogen atoms are omitted for clarity.

Molecular dynamics simulation was carried out using an open-source LAMMPS program package [27] as a free, powerful, and convenient tool. Mixed halide perovskite CH_3_NH_3_Pb(Cl_1−*x*_Br*_x_*)_3_ supercells of 4 × 4 × 4 size with randomly distributed Cl/Br-atoms in the X-sublattice and randomly oriented CH_3_NH_3_^+^ cations in the A-sublattice were generated for each studied composition, *x*. In order to cover the greater number of different random Cl/Br distributions and orientations of CH_3_NH_3_^+^ cations in the lattice, each supercell was independently generated 5 times. For clarity, an example of the supercell with an *x* = 0.5 composition, obtained after the simulation, is illustrated in Figure 2.

The interatomic potentials, cutoff distances, and other parameters were borrowed from [19,28] and summarized in Tables S2–S6. Simulation runs were performed under NPT-constant conditions (*T* = 298 K, *p*° = 100 kPa) using a Nose–Hoover integrator with Parrinello–Rahman barostat and 3 chains for the thermostat and barostat. The time step of the simulation was 2 fs, while the total time of the single simulation run was 4 ns. The atomic positions were dumped every 0.2 ns for further analysis. The values of the thermodynamic parameters after equilibration (occurred at timestamp of ca. 50 ps) were collected and averaged over the simulation time for the single run. The obtained time-averaged values of the five independently generated supercells corresponding to the same composition were compared with each other to test the effect of the distributions of Cl/Br and orientations of CH_3_NH_3_^+^. If such an influence was not identified, the five time-averaged values of the thermodynamic parameters were again averaged between each other.

## 3. Results and Discussion

### 3.1. Sample Characterization

The results of the chemical analysis are presented in Table S7. The measured and calculated mass fractions of the elements are in a good agreement with each other within the specified uncertainty limits.

**Figure 3:**
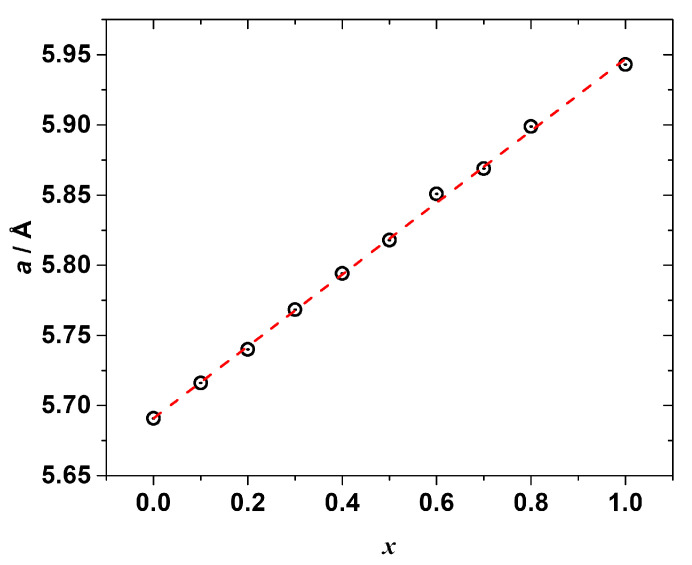
The refined cubic lattice parameter *a* of the synthesized mixed halide perovskites CH_3_NH_3_Pb(Cl_1−x_Br_x_)_3_ as a function of composition *x* (black circles), red dashed line represents a linear fitting.

The PXRD measurements (Figures S1–S10) confirmed the formation of the solid solutions. All the synthesized samples (except the one with *x* = 0.9) were found to be single phase with a cubic *Pm*-3*m* perovskite crystal structure similar to the end-members CH_3_NH_3_PbCl_3_ and CH_3_NH_3_PbBr_3_. The refined lattice constants are listed in Table S8 and shown in Figure 3 as a function of the chemical composition. As seen, the lattice parameter *a* is linearly correlated (*R*^2^ = 0.999) with the molar fraction of CH_3_NH_3_PbBr_3_, i.e., the Vegard’s law is fulfilled:

*a*(CH_3_NH_3_Pb(Cl_1−*x*_Br*_x_*)_3_) = (1 − *x*) · *a*(CH_3_NH_3_PbCl_3_) + *x* · *a*(CH_3_NH_3_PbBr_3_), (6)

*a*(CH_3_NH_3_Pb(Cl_1−*x*_Br*_x_*)_3_) = 5.69113 + 0.25605*x*. (7)

Despite reproducing the synthesis several times, we were unsuccessful in synthesizing the single-phase solid solution with *x* = 0.9; therefore, this sample was e*x*cluded from the further study. Since all the other solid solutions were synthesized via the same route, we may tentatively suggest that the solid solution with *x* = 0.9 possesses the lowest thermal stability among the studied samples and degrades under the chosen annealing conditions, which is why we did not obtain it as a single-phase compound. The analysis of its PXRD pattern revealed the presence of several peaks of an unidentified impurity phase (see Figure S11). In addition, the refined lattice parameter (see Table S8) of the main perovskite phase was significantly larger than the one expected from Vegard’s law, being closer to the lattice constant of the pure CH_3_NH_3_PbBr_3_.

### 3.2. Results of Solution Calorimetry

The measured solution enthalpies of all the studied samples in DMSO at *T* = (298.15 ± 0.04) K (expanded uncertainty, level of confidence 95%) are summarized in Table S9.

The mixing enthalpies of the solid solutions were calculated using the thermochemical cycle, involving the following processes (the subscript ‘s’ denotes the solid state of the material, the subscript ‘sol’ denotes the solute state of the material in DMSO):

CH_3_NH_3_PbCl_3 (298 K, s)_ → CH_3_NH_3_PbCl_3 (298 K, sol)_, Δ_sol_*H*_0,298_, (8)

CH_3_NH_3_PbBr_3 (298 K, s)_ → CH_3_NH_3_PbBr_3 (298 K, sol)_, Δ_sol_*H*_1,298_, (9)

CH_3_NH_3_Pb(Cl_1−*x*_Br*_x_*)_3 (298 K, s)_ → (1 − *x*) CH_3_NH_3_PbCl_3 (298 K, sol)_ + *x* CH_3_NH_3_PbBr_3 (298 K, sol)_, Δ_sol_*H*_*x*__,298_. (10)

Strictly speaking, to combine the above equations into the thermochemical cycles, for each solid solution, the final states of the solutes should be the same after dissolving either the end-members or a solid solution. Since the final concentrations of the solutions after the calorimetric experiments are quite low (do not exceed ~2 × 10^−3^ mol·kg^−1^), we neglected the compositional variation in the solution enthalpies and, therefore, operated them as the values corresponding to the conditions of an infinite dilution. Thus, combining Equations (8)–(10), along with their enthalpies, one can easily obtain the following expression for the standard mixing enthalpy at *T* = 298.15:

Δ_mi*x*_*H*°*_x_*_,298_ = (1 − *x*) · Δ_sol_*H*_0,298_ + *x* · Δ_sol_*H*_1,298_ − Δ_sol_*H_x_*_,298_, (11)

which corresponds to the formation of a solid solution from the end-members according to the reaction:

(1 − *x*) CH_3_NH_3_PbCl_3 (298 K, s)_ + *x* CH_3_NH_3_PbBr_3 (298 K, s)_ → CH_3_NH_3_Pb(Cl_1−*x*_Br*_x_*)_3 (298 K, s)_, Δ_mi*x*_*H*°*_x_*_,298_ (12)

**Figure 4:**
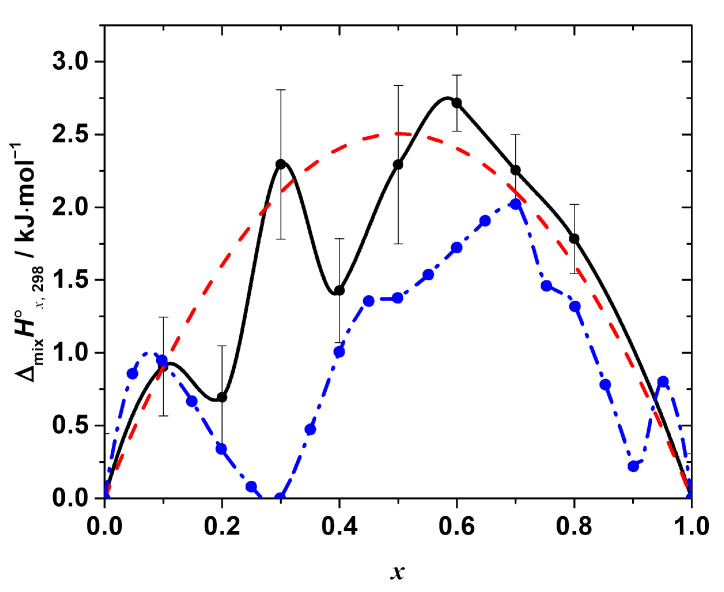
The standard mixing enthalpy of the solid solutions CH_3_NH_3_Pb(Cl_1−*x*_Br*_x_*)_3_ as a function of *x*. Black points—experimental values, obtained by the calorimetry at *T* = (298.15 ± 0.04) K, *p*° = (100 ± 4) kPa (expanded uncertainties, level of confidence 95%) in this work, black line—Akima spline interpolation of these points. Red dashed line represents the best fit of the regular solution model. Blue points—values, obtained in the molecular dynamics simulation experiment, described in [19] (reproduced from the graph in the original paper), blue dashed line—Akima spline interpolation of the data.

The as-calculated mixing enthalpies are given in Table 1 and shown in Figure 4.

As seen in Figure 4, the enthalpy of the mixing is positive over the entire range of the compositions with the maxima at *x* = 0.1, 0.3, and 0.6, i.e., the Δ_mi*x*_*H*°*_x_*_,298_-composition dependence apparently has a complex, non-monotonic nature. The positive mixing enthalpy indicates that the formation of the solid solutions is not an energetically favorable process, at least from the point of view of the enthalpy contribution to the Gibbs free energy change. Moreover, obviously, such simple models as, for example, the regular solution one:

Δ_mi*x*_*H*°*_x_*_,298_ = *a* · *x* · (1 − *x*), (13)

cannot fit the experimental data satisfactory within the estimated expanded uncertainty range of the experimental values. However, a rough estimation of the phase stability using that model may be performed, revealing interesting results. Fitting the obtained experimental data using Equation (13) results in the interaction parameter, ‘*a*’, equal to (10.02 ± 1.44) kJ·mol^−1^ (expanded uncertainty, 95% level of confidence; R^2^ = 0.81). Assuming the solution is regular, i.e., assuming a non-zero mixing enthalpy and ideal configurational mixing entropy, the upper solubility temperature *T*_c_ = (*a*/2R) [29] may be estimated as ~600 K. Therefore, within this approximation, the solid solutions are expected to be unstable at *T* < 600 K. That fact, obviously, disagrees with the experimental observations, since the solid solutions studied in this work were successfully synthesized at *T* = 423 K. Thus, the assumption of the regularity of the solid solutions of interest is inadequate, and the mixing entropy is not determined by the configurational contribution only. The magnitude of the mixing enthalpy is apparently quite high; therefore, the additional stabilizing contributions to the mixing entropy must be considered to estimate the phase stability of the solid solutions correctly. The nature of these contributions (vibrational, defect, or others) requires additional study.

The other important feature that we would like to point out is the complex, non-monotonic nature of the experimentally observed mixing enthalpy. The largest deviations of the experimental points (of the order of 0.5–1 kJ·mol^−1^, as compared to ~2.7 kJ·mol^−1^ as a maximum value of the mixing enthalpy) from the curve given by Equation (13) occur at *x* = 0.2 and 0.4. It is to be emphasized that all the obtained values of Δ_mi*x*_*H*°*_x_*_,298_ were reproducible, i.e., they result from the real sample behavior and not from some flaws in the experiments. Interestingly, the similar complex behavior of the mixing enthalpy resembling the one revealed in this work has been recently reported in [19] based on the results of the molecular dynamics simulation of the CH_3_NH_3_Pb(Cl_1−*x*_Br*_x_*)_3_ solid solutions. The Δ_mi*x*_*H*°*_x_*_,298_ calculated in [19] is also presented in Figure 4 for the sake of comparison. Both dependences of Δ_mi*x*_*H*°*_x_*_,298_ on the composition apparently share similar features, such as a non-monotonic nature with a pronounced sequence of minima and maxima, as described above. However, in the original work, the behavior of Δ_mi*x*_*H*°*_x_*_,298_ was neither discussed in detail nor compared with the experimental one because of the absence of the experimental measurements. Therefore, we performed a similar molecular dynamics simulation to get some insights on the local-level behavior of the CH_3_NH_3_Pb(Cl_1−x_Br_x_)_3_ solid solutions.

### 3.3. Molecular Dynamics Simulation Results

**Figure 5:**
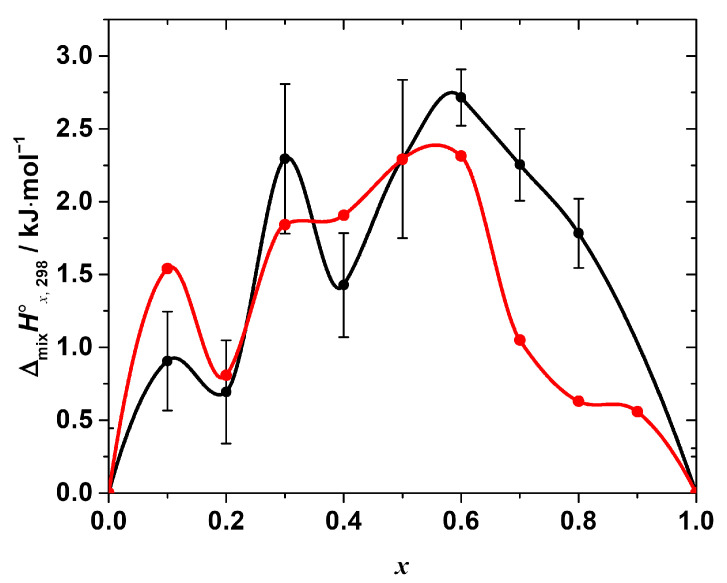
Standard mixing enthalpy of the solid solutions CH_3_NH_3_Pb(Cl_1−*x*_Br*_x_*)_3_ determined by calorimetry (black points) in comparison with the one calculated from the results of molecular dynamics simulation (red points). All the dependences are interpolated by Akima splines.

As described in Section 2, the molecular dynamics simulation was carried out in the whole range of the compositions. The average enthalpy for each composition of the solid solution was computed and the mixing enthalpy was calculated in the following way:

<Δ_mi*x*_*H*°*_x_*_,298_> = <*H_x_*_,298_> − [(1 − *x*) · <*H*_0,298_> + *x* · <*H*_1,298_>], (14)

where <*H*_0,298_> and <*H*_1,298_> are the average enthalpies of the end-members with *x* = 0 and 1, respectively, and <*H_x_*_,298_> is the average enthalpy of the particular mixed halide solid solution. The as-calculated values of <Δ_mi*x*_*H*°*_x_*_,298_> are shown in Figure 5 as a function of *x* in comparison with the experimental ones.

As seen from Figure 5, the overall Δ_mi*x*_*H*°*_x_*_,298_-composition dependence, calculated from the molecular dynamics simulation in this work, shows a surprisingly good agreement with that determined experimentally. The simulated mixing enthalpy also shows (a) positive values in the whole range of the compositions, and (b) a clear non-monotonic nature with an apparent minima at the compositions with *x* = 0.2, 0.4, and ~0.8. In addition, the calculated values are in a better agreement with the experimental ones in the Cl-rich region than in Br-rich one.

In general, the positive excess energy or enthalpy of mixing is expected when mixing the end-members with significantly different molar volumes [30,31,32,33], like in the case of the solid solutions CH_3_NH_3_Pb(Cl_1−*x*_Br*_x_*)_3_ (the end-members’ molar volumes differ by almost 14%). This mismatch results in the local elastic strains due to the distribution (supposedly random) of Cl and Br-anions with non-equal radii in the halide sublattice of the perovskite lattice. This is also accompanied by the variation in the chemical bonds’ (ionic, covalent, intermolecular, etc.) strength upon the formation of the solid solution from the end-members.

In the particular case of the mixed hybrid halide perovskites, there are several types of interactions which may be affected by the substitution. Moreover, variations in the elastic strain energy and of the strength of various interactions between the lattice species are most probably interdependent. For example, it is expected that the interionic Pb–X and X–X interactions may depend strongly both on the local coordination and distortions of the [PbX_6_] octahedra and on the overall lattice strain, induced by the sequential substitution of the anions in the X-sublattice.

Furthermore, the N–H···X hydrogen bonds between the ammonium’s hydrogen and halide atoms play a significant role in the energetics of the organic–inorganic halide perovskite lattice. In the case of mixed halide compounds, the halide substitution should lead to a coexistence of the energetically nonequivalent N–H···Cl and N–H···Br hydrogen bonds, considerably changing both the static and dynamic properties of the material [34,35]. Therefore, a complex interplay between the local strains and energetics of various interactions of the lattice species should lead to a nontrivial variation in the lattice energy and, hence, in the mixing enthalpy as well, as observed experimentally in this work.

**Figure 6:**
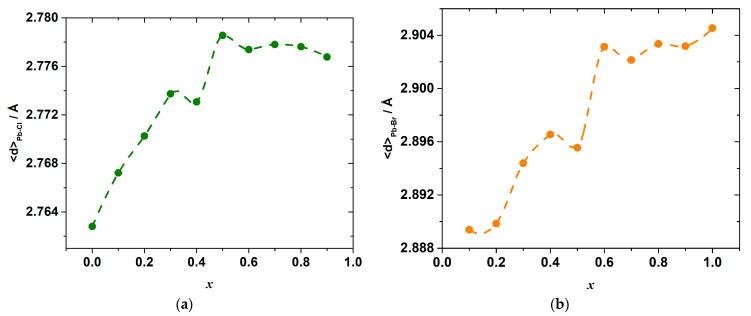
Average bond lengths Pb–Cl (**a**) and Pb–Br (**b**) as a function of composition *x* in CH_3_NH_3_Pb(Cl_1−x_Br_x_)_3_ as simulated by molecular dynamics.

**Figure 7:**
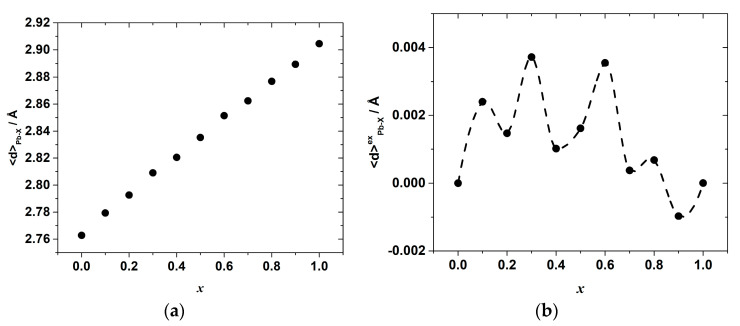
(**a**) The overall average Pb–X (X = Br, Cl) bond length and (**b**) its excess value as a function of composition, *x*, in CH_3_NH_3_Pb(Cl_1−x_Br_x_)_3_.

The results of the molecular dynamics simulations allow revealing the abovementioned local variations of the strain and interactions. Indeed, the change in the energy of the interionic interactions in the inorganic framework of the hybrid perovskite halide may be demonstrated, for example, through the observation of the variation in the Pb–X bond lengths. The calculated average Pb–Cl, <*d*>_Pb–Cl_, and Pb–Br, <*d*>_Pb–Br_, distances are shown in Figure 6. The overall mean Pb–X, <*d*>_Pb–X_, distance, and its excess value, which is defined as the deviation from the straight line between the <*d*>_Pb–Cl_ and <*d*>_Pb–Br_ in the end-members CH_3_NH_3_PbCl_3_ and CH_3_NH_3_PbBr_3_, is shown in Figure 7.

As seen in Figure 6, a substitution of Br for Cl ions progressively increases the mean Pb–Cl bond length as compared to <*d*>_Pb–Cl_ in the pure CH_3_NH_3_PbCl_3_ end-member, with a simultaneous shortening of the mean Pb–Br bond length with respect to <*d*>_Pb–Br_ in CH_3_NH_3_PbBr_3_. Therefore, the mixed-ligand octahedra [PbX_6_] in the solid solutions consist of elongated Pb–Cl bonds, which are expected to be less strong in comparison with ones in the [PbCl_6_] octahedra of CH_3_NH_3_PbCl_3_, and shortened Pb–Br bonds, which are expected to be stronger than those in the [PbBr_6_] octahedra of CH_3_NH_3_PbBr_3_. Interestingly, the bond lengths change strongly upon substituting Br for Cl ions only until the composition reaches *x* = 0.6. A further substitution leads only to an insignificant variation in the bond lengths. This may be because it is easier to substitute a smaller ion (Cl^−^) into a site that is normally occupied by a larger ion (Br^−^) than the other way around, as was noted before for the other solid solutions [31,32,33].

Despite this, it is likely that the Pb–X bond energies in the octahedra depend not only on the bond lengths, but also on the particular configuration of the octahedra and other bond characteristics, the total energy change upon the substitution is determined by the sum of both effects of the elongation and a shortening of the Pb–Cl and Pb–Br bonds, respectively. In a general case, this complex relationship between the energy and interionic distance hinders the direct comparison between these two properties, so their compositional dependences are not expected to mimic one another exactly. Nevertheless, the complex shape of both dependences shown in Figure 6 possesses a series of minima and maxima at concentrations close to the locations of the extrema on the Δ_mi*x*_*H*°*_x_*_,298_ vs. *x* dependence.

This correlation can be seen more clearly on the excess bond length, <*d*>^e*x*^_Pb–X_, dependence plotted in Figure 7. The overall average Pb–X bond length, in turn, gradually increases with *x*, as seen in Figure 7a, whereas the excess bond length, <*d*>^e*x*^_Pb–X_, shows some oscillations again with characteristic minima at *x* = 0.2, 0.4 and 0.7–0.9, as demonstrated in Figure 7b.

The values of <*d*>^e*x*^_Pb–X_ are positive in the wide range of compositions up to *x*~0.8, which indicates the additional net increase in the Pb–X bond length and agrees with the observed positive e*x*cess enthalpy. Only in the range of *x* = 0.8–1 is there a slight shortening of the mean Pb–X bond length. The minima and maxima of the excess average Pb–X bond length suggest that from the viewpoint of the accommodation of the lattice strain, there are some more distorted (and, therefore, energetically less favored) compositions corresponding to the <*d*>^e*x*^_Pb__−X_ maxima, and some less distorted ones corresponding to the minima of the <*d*>^e*x*^_P__b−X_ compositional dependence. The fact that the shape of the <*d*>^e*x*^_Pb__−X_ vs. *x* dependence resembles, although not exactly, that of the mixing enthalpy, indicates that the observed complex compositional dependence of the interionic Pb–X bond lengths, which points to a complex distribution of local elastic strains, may be one of the reasons for the nontrivial compositional behavior of the mixing enthalpy in the CH_3_NH_3_Pb(Cl_1−x_Br_x_)_3_ system.

Another point concerns the important role hydrogen bonds play in the energetics of the hybrid halide perovskite lattice. Unfortunately, it is impossible to directly extract hydrogen bond energies from the molecular dynamic simulation because of the complex nature of the used force-field. The only characteristic which can be estimated instead is the hydrogen bond length. Then, using some appropriate model relating the hydrogen bond energy to the bond length allows (although indirectly) computing a rough estimate of the compositional variation in the overall energy of the hydrogen bonds.

In this work, we estimated the interaction energy that is identified with the hydrogen bond energy (see the detailed definition in [36]) from the model potential energy function, given in [36], and based on ab initio calculations. The model suggested in [36] describes the hydrogen bond energy, *E*_HB_, as being proportional to *r*^−3.8^, where *r* is an equilibrium distance between a hydrogen atom and an acceptor. The hydrogen bond energies were estimated using the appropriate constants, given in the original paper [36], for the nitrogen atom as a donor and Cl (or Br) as an acceptor of the hydrogen bonds.

**Figure 8:**
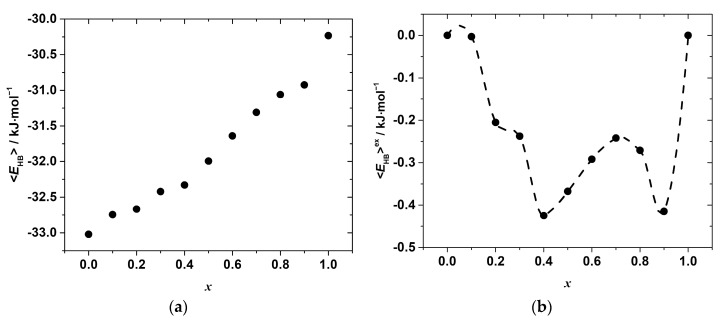
Compositional dependences of the (**a**) mean N-H···X hydrogen bond energy per mole of CH_3_NH_3_Pb(Cl_1−x_Br_x_)_3_ solid solution (**b**) excess N-H···X hydrogen bond energy per mole of the solid solution.

The calculations were carried out in the following way. First, the pairs of the H and X atoms, for which the distance is less than the sum of the Van der Waals radii of the elements, were identified. They were assumed to be bonded by hydrogen bonds. Then, for each pair of the atoms, the hydrogen bond energy was calculated using the potential function mentioned above. The computed energies were averaged for each composition and reduced to the energy per mole of the solid solution, <*E*_HB_>. Similarly to the mixing enthalpy (Equation (14)) and the mean Pb–X bond length, the excess hydrogen bond energies were calculated as follows:

<*E*_HB_>^e*x*^ = <*E*_HB_>*_x_ −* [(1 − *x*) · <*E*_HB_>_0_ + *x* · <*E*_HB_>_1_], (15)

where <*E*_HB_>*_0_* and <*E*_HB_>*_1_* are the average hydrogen bond energies per mole of the end-members with composition *x* in CH_3_NH_3_Pb(Cl_1−x_Br_x_)_3_ equal to 0 and 1, respectively. The compositional dependences of the as-estimated <*E*_HB_> and <*E*_HB_>^e*x*^ are shown in Figure 8.

As seen, the hydrogen bonds become weaker with the increase in the concentration of bromide, *x,* which is expected due to the differences in the electronegativity of Cl and Br. However, as in the case of the Pb–X distances, the variation in <*E*_HB_>*_x_* with the composition is far from linear and shows some irregular behavior, which is more clearly revealed by the compositional dependence of the excess hydrogen bond energy presented in Figure 8b. Negative deviations from linearity in the whole range of the compositions suggest a formation of stronger hydrogen bonds in the solid solutions as compared to the simple mixture of chloride and bromide end-members. Furthermore, several minima indicate the formation of stronger hydrogen bonds and agree with the minima on the Δ_mi*x*_*H*°*_x_*_,298_ vs. *x,* and <*d*>^e*x*^_Pb–X_ vs. *x* curves. This seems to indicate that the formation of stronger hydrogen bonds may compensate, at least partially, the energy penalty due to the lattice strain caused by the substitution.

## 4. Conclusions

The results of the molecular dynamics simulation indicate that all the lattice interactions in CH_3_NH_3_Pb(Cl_1−*x*_Br*_x_*)_3_ considered above share one distinctive feature, namely, non-monotonic variations with the degree of the halide substitution in the X-sublattice. These variations correlate (at least partially) with the experimentally observed non-monotonic change in the mixing enthalpy. Therefore, there is a comple*x* interplay between the local lattice strain, the energetics of the inorganic framework of the perovskite, and the hydrogen bonds between the organic cation and inorganic framework, which is responsible for the observed nontrivial compositional dependence of the mixing enthalpy of the CH_3_NH_3_Pb(Cl_1−*x*_Br*_x_*)_3_ mixed halide solid solutions.

At the moment, thermodynamic studies of the perovskite halide solid solutions are highly scarce. However, based on the results obtained in this work, several important points may be outlined. First, the formation of the studied solid solutions is not a favorable process from the viewpoint of the positive enthalpy contribution to the Gibbs free energy change. Second, the ideal configurational mixing entropy itself is not enough to compensate for the observed positive formation enthalpy and make the Gibbs free energy of mixing negative, at least at *T* < 600 K. Therefore, additional contributions to the mixing entropy are inevitably present with a magnitude, at least, comparable to the configurational one. The nature of these contributions requires additional study. All in all, this suggests that making solid solutions does not always automatically imply obtaining the materials with an increased intrinsic stability.

What is more, the presence of the minima/maxima on the excess enthalpy curve may lead to similar ones on the excess Gibbs free energy vs. the composition dependence, which indicates a thermodynamically favorable phase separation in the solid solutions CH_3_NH_3_Pb(Cl_1−*x*_Br*_x_*)_3_ at room temperature. However, if such a phase separation is not observed experimentally, it means that either kinetic limitations or some additional stabilizing thermodynamic factors, such as excess entropy, should be considered. Taking into account the complex shape of the compositional dependence of the mixing enthalpy, a similar complexity may be expected in the case of the excess entropy as well. Thus, a further experimental study is needed to gain a solid understanding of the thermodynamics of mixed halide perovskite systems.

## Figures and Tables

**Table 1:** The standard mixing enthalpies, Δ_mi*x*_*H*°*_x_*_,298_, in the CH_3_NH_3_PbCl_3_–CH_3_NH_3_PbBr_3_ system at *T* = 298.15 K, *p*° = 100 kPa, calculated from the solution enthalpies by Equation (11).

Formula	*x*	Δ_mi*x*_*H*°*_x__,_*_298_/kJ·mol^−1^ *
CH_3_NH_3_Pb(Cl_0.9_Br_0.1_)_3_	0.1	0.91 ± 0.34
CH_3_NH_3_Pb(Cl_0.8_Br_0.2_)_3_	0.2	0.69 ± 0.35
CH_3_NH_3_Pb(Cl_0.7_Br_0.3_)_3_	0.3	2.29 ± 0.51
CH_3_NH_3_Pb(Cl_0.6_Br_0.4_)_3_	0.4	1.43 ± 0.36
CH_3_NH_3_Pb(Cl_0.5_Br_0.5_)_3_	0.5	2.29 ± 0.54
CH_3_NH_3_Pb(Cl_0.4_Br_0.6_)_3_	0.6	2.72 ± 0.19
CH_3_NH_3_Pb(Cl_0.3_Br_0.7_)_3_	0.7	2.25 ± 0.25
CH_3_NH_3_Pb(Cl_0.2_Br_0.8_)_3_	0.8	1.78 ± 0.24

* The numbers following the symbol ± correspond to the values of the expanded uncertainties *U*_c_ = *k*·*u*_c_ determined from a combined standard uncertainty *u*_c_ and a coverage factor *k* = 2, corresponding to 95% level of confidence. Expanded uncertainties *U*_c_(*T*) = 0.04 K, *U*_c_(*p*°) = 4 kPa.

## Data Availability

The reported data are available from the corresponding authors on a reasonable request.

## Supplementary Materials

The following supporting information can be downloaded at: https://www.mdpi.com/article/10.3390/ma15217675/s1. Table S1. reagents used in this work; Table S2. Parameters of the Buckingham potential (Equation (S1)), used in this work and taken from [19]; Table S3. Parameters of the Lennard-Jones potential (Equation (S2)), used in this work; Table S4. Parameters of the harmonic bonds energy (Equation (S3)), used in this work and taken from [28]; Table S5. Parameters of the harmonic angles energy (Equation (S4)), used in this work and taken from [28]; Table S6. Partial charges, *q*, used in this work and taken from [19]; Table S7. Calculated and measured weight fractions of the elements in the synthesized solid solutions; Figures S1–S11: Powder XRD pattern of CH_3_NH_3_Pb(Cl_1−*x*_Br*_x_*)_3_ after Rietveld refinement; Table S8. Lattice parameters of the obtained solid solutions at *T* = 298 K, refined by Rietveld analysis of the PXRD patterns; and Table S9. The solution enthalpies of the solid solutions samples in dimethylsulfoxide (DMSO) at *T* = 298.15 K, *p*° = 100 kPa.
