# Supplementary material for: Thermochemical Study of CH3NH3Pb(Cl1−xBrx)3 Solid Solutions
Source: Materials (Basel). 2022 Nov 1;15(21):7675. doi: 10.3390/ma15217675 (PMC9657867; doi:10.3390/ma15217675)
Supplement: Supplementary file 1 [file materials-15-07675-s001.zip › materials-1974350-supplementary.pdf]

Thermochemical study of $CH_3NH_3Pb(Cl_{1-x}Br_x)_3$ solid solutions

Maxim Mazurin, Angelika Shelestova, Dmitry Tsvetkov, Vladimir Sereda, Ivan Ivanov, Dmitry Malyshkin, Andrey Zuev *

Institute of Natural Sciences and Mathematics, Ural Federal University, 19 Mira St., Ekaterinburg 620002, Russia

* Correspondence: andrey.zuev@urfu.ru

Supplementary material

Table S1. Reagents used in this work

Compound	Source	Mass fraction purity	Analysis method
$PbCl_2$	Vekton, Russia	0.995	stated by the supplier
$Pb(CH_3COO)_2 \cdot 3H_2O$	Vekton, Russia	0.995	
HBr, water solution, 46 mass %	"ORT Himreactivy", Russia	0.998	
HCl, water solution, 38 mass %	Ormet, Russia	0.9995	
CH_3NH_2, water solution, 38.5%	Ormet, Russia	0.999	
Dimethylsulfoxide (DMSO)	Vekton	0.995	stated by the supplier
KCl	Lanhit, Russia	0.99998	
CH_3NH_3Cl	Synthesis	0.99	XRD[a]
CH_3NH_3Br	Synthesis	0.99	
$PbBr_2$	Synthesis	0.99	
$CH_3NH_3Pb(Cl_{1-x}Br_x)_3$	Synthesis	0.99	XRD, elemental analysis

[a] X-ray diffraction measurements

Molecular dynamics simulation details

Following the [19] and [28], intermolecular interactions in this work were described using the combination of the well-known potential functions, namely:

Buckingham potential:

$$U(r) = Ae^{-r\rho} - \frac{C}{r^6} \quad \text{(S1)}$$

and Lennard-Jones potential:

$$U(r) = 4\varepsilon\left(\left(\frac{\sigma}{r}\right)^{12} - \left(\frac{\sigma}{r}\right)^{6}\right) \quad \text{(S2)}$$

Parameters, used in the calculations are listed below in Tables S2 and S3.

Table S2. Parameters of the Buckingham potential (Equation (S1)), used in this work and taken from [19]

**Pair**	**A / kcal·mol^{-1}**	**ρ / Å^{-1}**	**C / Å^6·kcal·mol^{-1}**
Pb-Pb	70359906	0.131258	0
Pb-Br	103496	0.30368	0
Pb-Cl	103496	0.2917	0
Pb-C	32690390	0.150947	0
Pb-N	32690390	0.150947	0
Br-Br	22793	0.42961	696.9495
Br-Cl	22793	0.4126616	696.9495
Br-C	112936	0.323208	0
Br-N	112936	0.323208	0
Cl-Cl	22793	0.3963	696.9495
Cl-C	112936	0.3104578	0
Cl-N	112936	0.3104578	0

Table S3. Parameters of the Lennard-Jones potential (Equation (S2)), used in this work*

**Pair**	**ε / kcal·mol^{-1}**	**σ / Å**
Pb-H_1[a]	0.0140	2.26454
Pb-H_2[a]	0.0140	2.70999
Br-H_1[a]	0.574	2.5957
Br-H_2[a]	0.574	2.9260
Cl-H_1[a]	0.574	2.4932
Cl-H_2[a]	0.574	2.8105
C-H_1[a]	0.0414	2.23440
C-H_2[a]	0.0414	2.6798
N-H_1[a]	0.0517	2.1595

N-H_2[a]	0.0517	1.069
C-C[b]	0.1094	3.39970
C-N[b]	0.1364	3.32480
H_1-H_2[b]	0.0157	1.51450
H_1-H_1[b]	0.0157	1.06910
H_2-H_2[b]	0.0157	1.96
N-N[b]	0.17	3.25

[*] H_1 denotes the –NH_3 hydrogen atom, H_2 denotes the –CH_3 one

[a] constants were taken from [19] (supplementary)

[b] constants were taken from [28] (supplementary)

The bonds and angles of methylammonium molecular ions were treated within the simple harmonic force-field model, where the energy, E equals:

$$E_{bond} = K(r - r_0)^2 \quad \text{(S3)}$$

$$E_{angle} = K(\theta - \theta_0)^2 \quad \text{(S4)}$$

for the bonds and angles respectively. r is interatomic distance in the molecule, θ is the angle. Force-field constants, used in the calculations and taken from [28] are listed below in the Tables S4 and S5.

Table S4. Parameters of the harmonic bonds energy (Equation (S3)), used in this work and taken from [28]*

**Bond**	***K* / kcal·Å^{-2}**	**r_0 / Å**
C-N	587.2	1.499
C-H_2	677.4	1.091
N-H_1	738.0	1.033

[*] H_1 denotes the –NH_3 hydrogen atom, H_2 denotes the –CH_3 one

Table S5. Parameters of the harmonic angles energy (Equation (S4)), used in this work and taken from [28]*

**Angle**	***K* / kcal·°$^{-2}$**	**θ / °**
N-C-H_2	98.0	107.91
H_1-N-C	92.4	110.11
H_2-C-H_2	78.0	110.74
H_1-N-H_1	81.0	108.11

[*] H_1 denotes the –NH_3 hydrogen atom, H_2 denotes the –CH_3 one

Table S6. Partial charges, *q*, used in this work and taken from [19]*

**Atom**	***q***
Pb	+2.03
Cl, Br	−1.13
C	+0.771
N	−1.1
H_1	+0.54
H_2	+0.023

* H_1 denotes the $–NH_3$ hydrogen atom, H_2 denotes the $–CH_3$ one

Table S7. Calculated and measured weight fractions of the elements in the synthesized solid solutions*

**Formula**	**C**		**H**		**N**	
	**Calc.**	**Meas.**	**Calc.**	**Meas.**	**Calc.**	**Meas.**
$CH_3NH_3PbCl_3$	0.035	0.039	0.018	0.018	0.041	0.042
$CH_3NH_3PbCl_{2.7}Br_{0.3}$	0.034	0.036	0.017	0.016	0.039	0.039
$CH_3NH_3PbCl_{2.1}Br_{0.9}$	0.031	0.033	0.016	0.015	0.036	0.037
$CH_3NH_3PbCl_{1.8}Br_{1.2}$	0.030	0.031	0.015	0.015	0.035	0.036
$CH_3NH_3PbCl_{1.5}Br_{1.5}$	0.029	0.030	0.015	0.013	0.034	0.036
$CH_3NH_3PbCl_{1.2}Br_{1.8}$	0.028	0.029	0.014	0.013	0.033	0.034
$CH_3NH_3PbCl_{0.9}Br_{2.1}$	0.027	0.028	0.014	0.013	0.032	0.033
$CH_3NH_3PbBr_3$	0.025	0.027	0.013	0.012	0.029	0.030

*Expanded uncertainty of weight fractions determination U_c = 0.003 ($U_c = k \cdot u_c$ determined from a combined standard uncertainty u_c and a coverage factor k = 2, corresponding to 95% level of confidence)

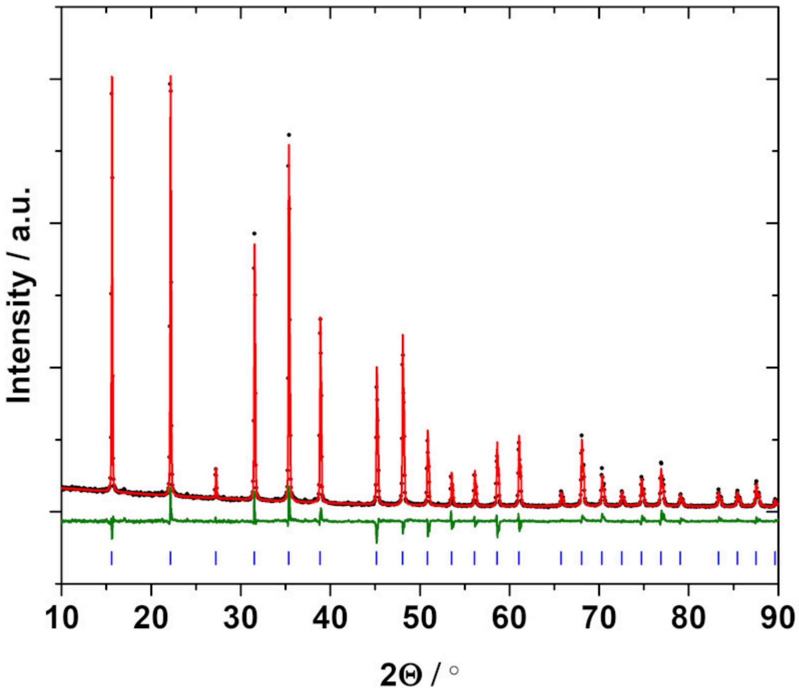

Figure S1. Powder XRD pattern of $CH_3NH_3PbCl_3$ (x = 0) after Rietveld refinement (R_w =8.01 %). Black points – observed pattern, red line – calculated pattern, green line – difference, blue ticks – Bragg's positions.

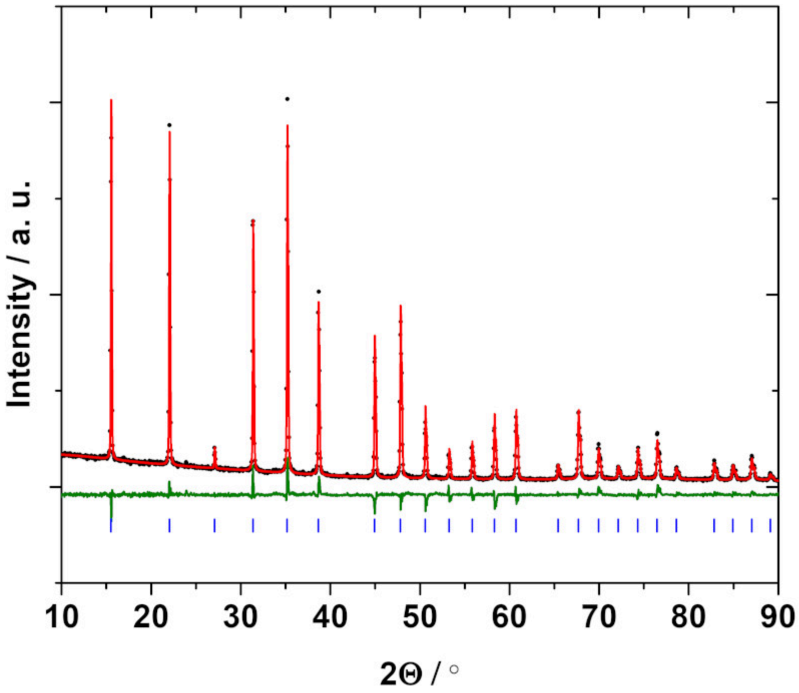

Figure S2. Powder XRD pattern of $CH_3NH_3PbCl_{2.7}Br_{0.3}$ (x = 0.1) after Rietveld refinement (R_w =7.32 %). Black points – observed pattern, red line – calculated pattern, green line – difference, blue ticks – Bragg's positions.

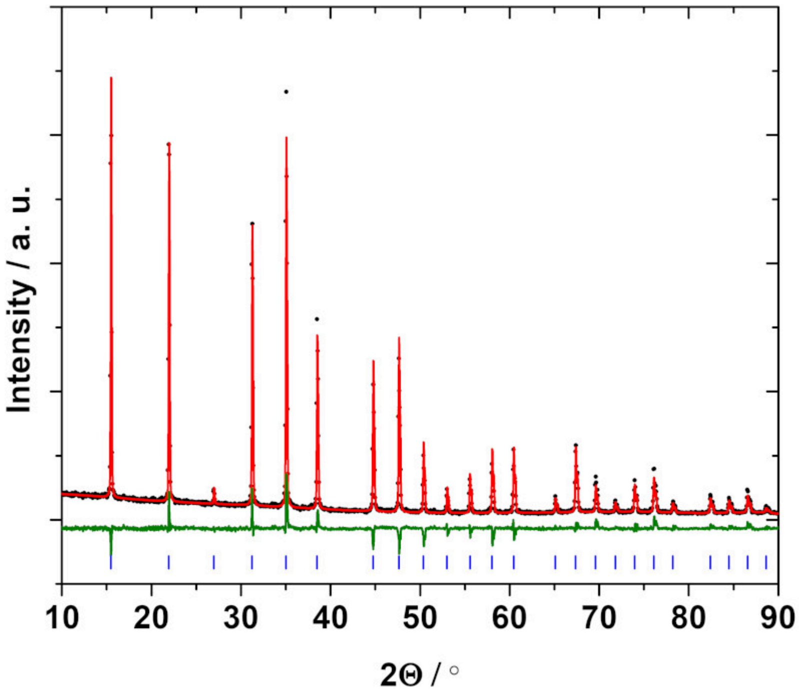

Figure S3. Powder XRD pattern of $CH_3NH_3PbCl_{2.4}Br_{0.6}$ ($x = 0.2$) after Rietveld refinement (R_w =9.25 %). Black points – observed pattern, red line – calculated pattern, green line – difference, blue ticks – Bragg's positions.

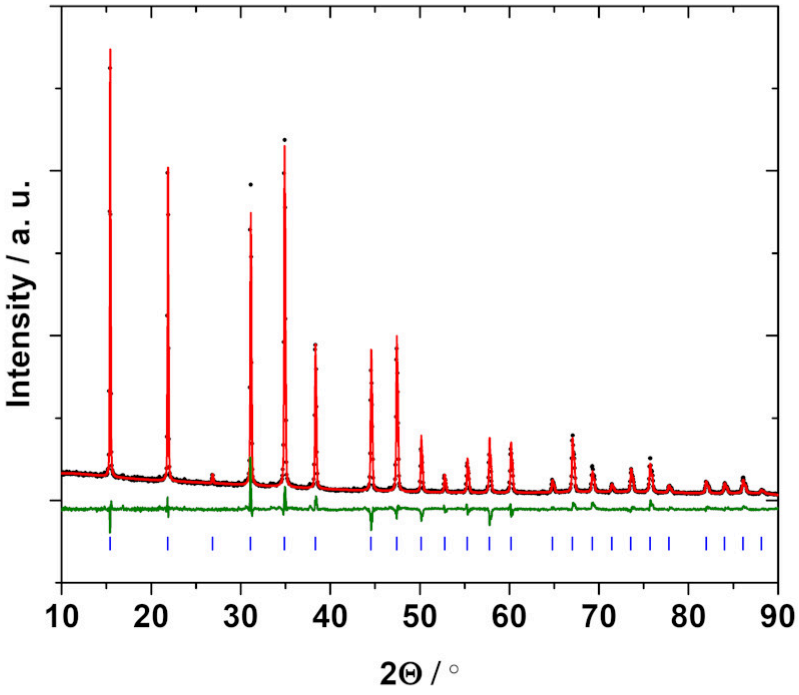

Figure S4. Powder XRD pattern of $CH_3NH_3PbCl_{2.1}Br_{0.9}$ ($x = 0.3$) after Rietveld refinement (R_w =7.47 %). Black points – observed pattern, red line – calculated pattern, green line – difference, blue ticks – Bragg's positions.

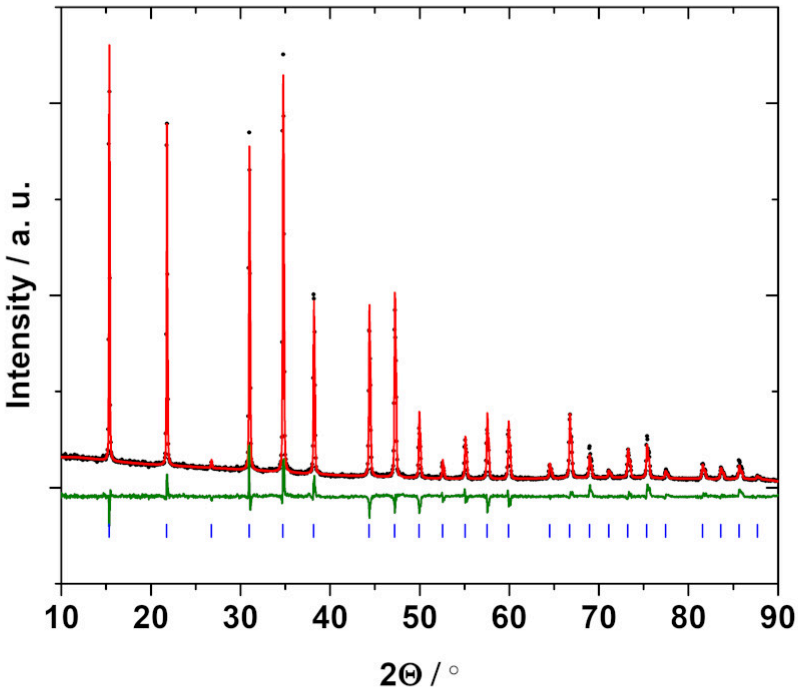

Figure S5. Powder XRD pattern of $CH_3NH_3PbCl_{1.8}Br_{1.2}$ ($x = 0.4$) after Rietveld refinement (R_w =8.01 %). Black points – observed pattern, red line – calculated pattern, green line – difference, blue ticks – Bragg's positions.

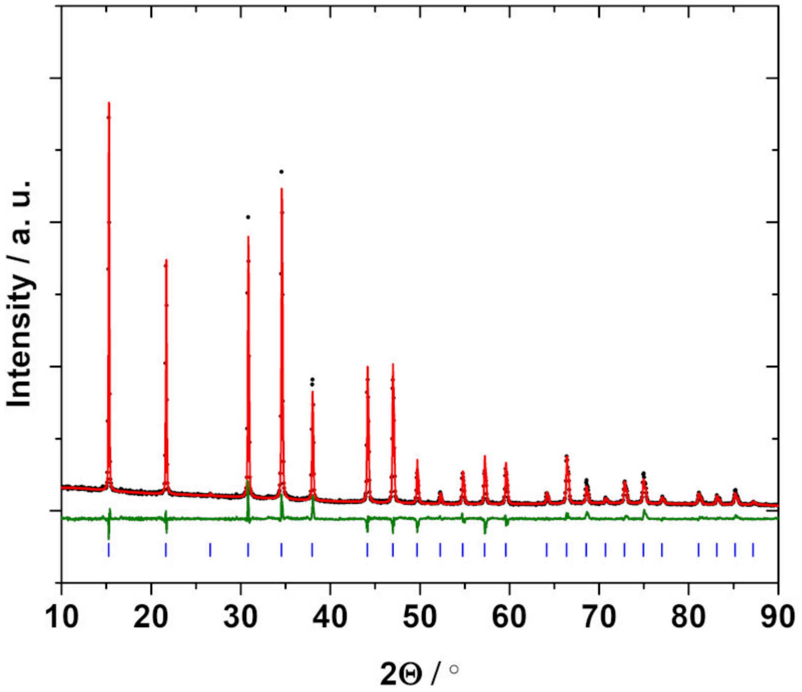

Figure S6. Powder XRD pattern of $CH_3NH_3PbCl_{1.5}Br_{1.5}$ ($x = 0.5$) after Rietveld refinement (R_w =7.64 %). Black points – observed pattern, red line – calculated pattern, green line – difference, blue ticks – Bragg's positions.

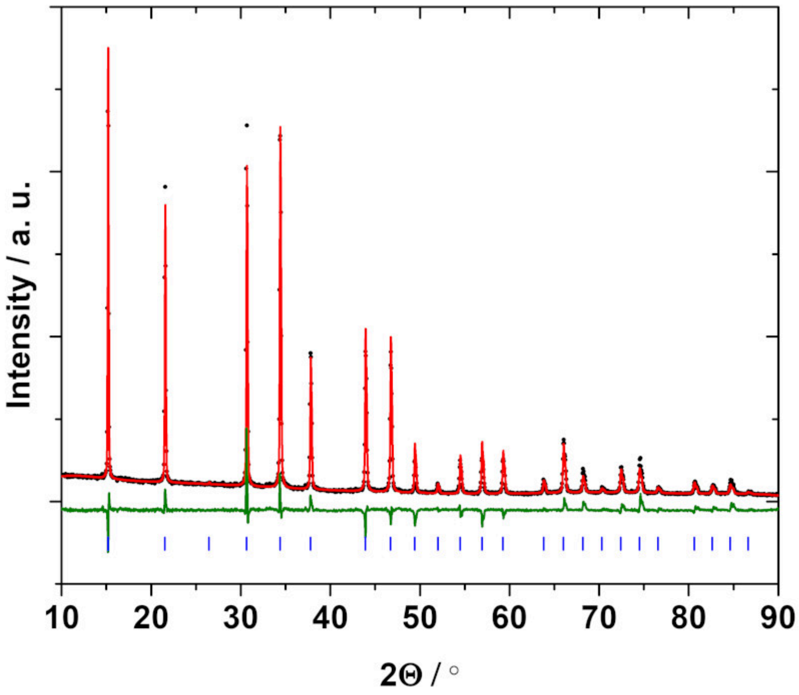

Figure S7. Powder XRD pattern of $CH_3NH_3PbCl_{1.2}Br_{1.8}$ ($x = 0.6$) after Rietveld refinement (R_w =9.3 %). Black points – observed pattern, red line – calculated pattern, green line – difference, blue ticks – Bragg's positions.

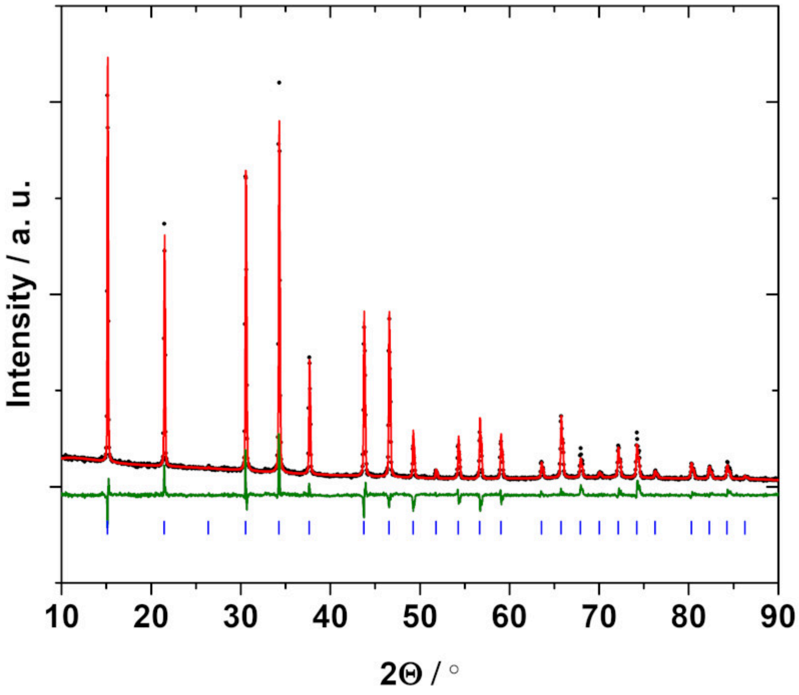

Figure S8. Powder XRD pattern of $CH_3NH_3PbCl_{0.9}Br_{2.1}$ ($x = 0.7$) after Rietveld refinement (R_w =8.57 %). Black points – observed pattern, red line – calculated pattern, green line – difference, blue ticks – Bragg's positions.

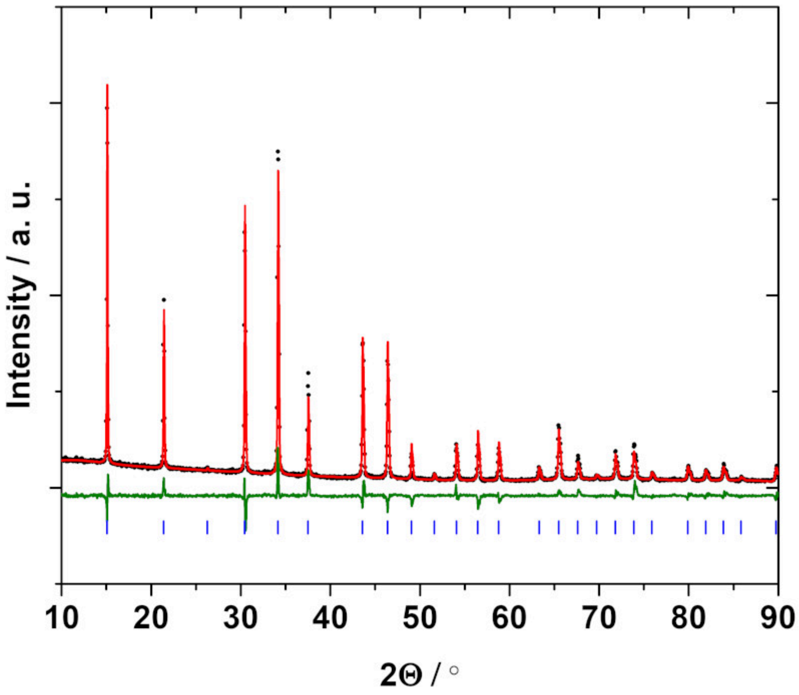

Figure S9. Powder XRD pattern of $CH_3NH_3PbCl_{0.6}Br_{2.4}$ ($x = 0.8$) after Rietveld refinement (R_w =9.13 %). Black points – observed pattern, red line – calculated pattern, green line – difference, blue ticks – Bragg's positions.

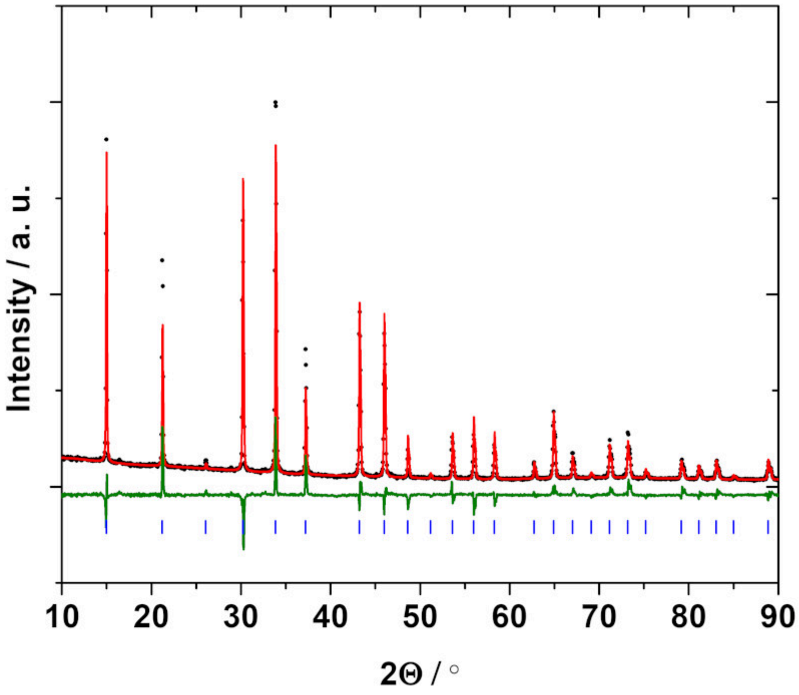

Figure S10. Powder XRD pattern of $CH_3NH_3PbBr_3$ ($x = 1$) after Rietveld refinement (R_w =11.91 %). Black points – observed pattern, red line – calculated pattern, green line – difference, blue ticks – Bragg's positions.

Table S8. Lattice parameters of the obtained solid solutions at $T = 298$ K, refined by Rietveld analysis of the PXRD patterns*

**Formula**	***x***	***a* / Å**
$CH_3NH_3PbCl_3$	0	5.6909
$CH_3NH_3PbCl_{2.7}Br_{0.3}$	0.1	5.7161
$CH_3NH_3PbCl_{2.4}Br_{0.6}$	0.2	5.7400
$CH_3NH_3PbCl_{2.1}Br_{0.9}$	0.3	5.7684
$CH_3NH_3PbCl_{1.8}Br_{1.2}$	0.4	5.7942
$CH_3NH_3PbCl_{1.5}Br_{1.5}$	0.5	5.8180
$CH_3NH_3PbCl_{1.2}Br_{1.8}$	0.6	5.8509
$CH_3NH_3PbCl_{0.9}Br_{2.1}$	0.7	5.8689
$CH_3NH_3PbCl_{0.6}Br_{2.4}$	0.8	5.8989
$CH_3NH_3PbCl_{0.3}Br_{2.7}$	0.9	5.9359
$CH_3NH_3PbBr_3$	1	5.9429

*Expanded uncertainty of lattice parameters determination $U_c(a) = 0.0004$ ($U_c = k \cdot u_c$ determined from a combined standard uncertainty u_c and a coverage factor $k = 2$, corresponding to 95% level of confidence). Expanded uncertainty $U_c(T) = 3$ K

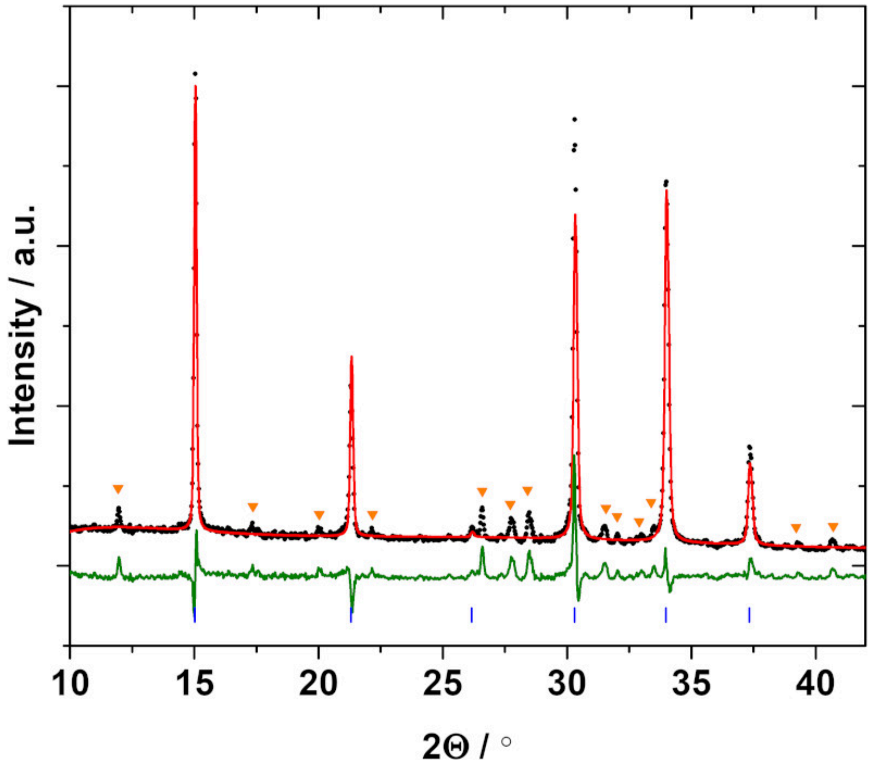

Figure S11. Rietveld refinement of PXRD pattern of the sample with probable composition $CH_3NH_3PbCl_{0.3}Br_{2.7}$ (x = 0.9). Black circles represents observed points, red line – calculated pattern, green line – difference, blue ticks – Bragg's positions; orange triangles highlight peaks of the unidentified impurity.

Table S9. The solution enthalpies of the solid solutions samples in dimethylsulfoxide (DMSO) at T = 298.15 K, $p°$ = 100 kPa; m represents the final molality of the solution, $\Delta_{sol}H_{298}$ represents the molar enthalpy of solution*

**Formula**	***x***	***m* × 10³ / mol·kg⁻¹**	**$\Delta_{sol}H_{298}$ / kJ·mol⁻¹**
$CH_3NH_3PbCl_3$	0	2.000 ± 0.004	–22.17 ± 0.31
$CH_3NH_3PbCl_{2.7}Br_{0.3}$	0.1	2.001 ± 0.004	–25.22 ± 0.19
$CH_3NH_3PbCl_{2.4}Br_{0.6}$	0.2	2.001 ± 0.003	–27.16 ± 0.25
$CH_3NH_3PbCl_{2.1}Br_{0.9}$	0.3	2.001 ± 0.003	–30.90 ± 0.46
$CH_3NH_3PbCl_{1.8}Br_{1.2}$	0.4	2.000 ± 0.006	–32.18 ± 0.29
$CH_3NH_3PbCl_{1.5}Br_{1.5}$	0.5	2.001 ± 0.003	–35.19 ± 0.51
$CH_3NH_3PbCl_{1.2}Br_{1.8}$	0.6	2.000 ± 0.004	–37.76 ± 0.07

$CH_3NH_3PbCl_{0.9}Br_{2.1}$	0.7	2.004 ± 0.003	–39.45 ± 0.17
$CH_3NH_3PbCl_{0.6}Br_{2.4}$	0.8	2.002 ± 0.003	–41.12 ± 0.15
$CH_3NH_3PbBr_3$	1	2.001 ± 0.003	–43.63 ± 0.22

*The numbers following the symbol ± correspond to the values of the expanded uncertainties $U_c = k \cdot u_c$ determined from a combined standard uncertainty u_c and a coverage factor $k = 2$, corresponding to 95% level of confidence. Expanded uncertainties $U_c(T) = 0.04$ K, $U_c(p°) = 4$ kPa
